# Association of acne, hirsutism, androgen, anxiety, and depression on cognitive performance in polycystic ovary syndrome: A cross-sectional study

**DOI:** 10.18502/ijrm.v18i12.8026

**Published:** 2020-12-21

**Authors:** Saeideh Mehrabadi, Shahideh Jahanian Sadatmahalleh, Anoshirvan Kazemnejad, Ashraf Moini

**Affiliations:** ^1^Department of Reproductive Health and Midwifery, Faculty of Medical Sciences, Tarbiat Modares University, Tehran, Iran.; ^2^Department of Biostatistics, Faculty of Medical Sciences, Tarbiat Modares University, Tehran, Iran.; ^3^Breast Disease Research Center (BDRC), Tehran University of Medical Sciences, Tehran, Iran.; ^4^Department of Obstetrics and Gynecology, Arash Women's Hospital, Tehran University of Medical Sciences, Tehran, Iran.; ^5^Department of Endocrinology and Female Infertility, Reproductive Biomedicine Research Center, Royan Institute for Reproductive Biomedicine, ACECR, Tehran, Iran.

**Keywords:** PCOS, Cognitive function, Androgen, Depression and anxiety.

## Abstract

**Background:**

While polycystic ovary syndrome (PCOS) is often associated with psychological distress, its most frequent clinical characteristics include acne, hirsutism and increased level of androgen hormones.

**Objective:**

To evaluate the level of depression and anxiety, hirsutism, acne, and level of androgen hormones in PCOS and control group and its association with cognitive function.

**Materials and Methods:**

This cross-sectional study was conducted on 53 women with PCOS and 50 healthy women as a control group. Data were collected using a questionnaire including the samples' demographic information, clinical features, clinical findings of hyperandrogenism, and the Beck Depression and Anxiety questionnaire. In addition, the acne and hirsutism levels of the subjects were evaluated using the global acne grading system and the Ferriman-Gallwey scoring system, respectively. The Montreal Cognitive Assessment (MoCA) is a screening test for cognitive impairment that covers major cognitive domains.

**Results:**

A significant difference was found between the two groups in the mean levels of acne, hirsutism, total testosterone, free androgen index, depression, and anxiety. However, some mean values of the MoCA were lower in the women of case group compared to the control group. Additionally, a significant difference was observed between the two groups in the domains of visual-spatial ability (p = 0.009), executive function (p = 0.05), attention (p = 0.03), and total MoCA scores (p = 0.002).

**Conclusion:**

The PCOS women demonstrated significantly lower performance on the tests of executive function, attention, and visual-spatial function than the healthy control women.

## 1. Introduction

Polycystic ovary syndrome (PCOS) can be diagnosed in a woman by the presence of at least two of the following three symptoms: 1) clinical and/or biochemical hyperandrogenism, 2) oligomenorrhea/amenorrhea, and 3) polycystic sonographic view in ovaries (1). It is the most common hormonal disorder in women of childbearing age, the prevalence of which is about 16.6% according to the Rotterdam criteria (2, 3). The symptoms of PCOS include oligomenorrhea, amenorrhea, obesity, hirsutism, infertility, acne, and anovulation, which can lead to anxiety, depression, sexual dysfunction, and social maladjustment (4) as well as a reduced quality of life, as indicated by several reports (5, 6). A besides, a large body of research now suggests that acne (7, 8), hirsutism, and androgen hormone levels (1, 9, 10) as well as depression and anxiety (11) can change the cognitive function.

The purpose of the current study was to evaluate the level of depression and anxiety, hirsutism, acne, and level of androgen hormones in PCOS group and its association with cognitive function compared to the control group.

## 2. Materials and Methods

### Participants

This cross-sectional study was conducted on women with PCOS, who attended the Gynecology Clinic in Arash Hospital in Tehran, Iran, from November 2015 to February 2016 in Tehran, Iran. Fifty-three women with a confirmed diagnosis of PCOS. Through the Rotterdam criteria (12), and 50 healthy women with normal bleeding were include and compared. The control group was matched in terms of age, education level, employment status, parity, marital status, height, and weight. After explaining the study objectives, a written approved informed consent was obtained from all participants. The inclusion criteria of the study were as follows: 1) age: 18-40 yr, 2) lack of recognized mental illness based on medical records and self-reports of the participants, 3) minimum literacy level of primary, 4) not using anti-androgen drugs, oral contraceptives, or any hormone therapy to treat the symptoms of PCOS within one month prior to recruitment, 5) the absence of stressful events in the last three months, 6) no chronic illnesses such as diabetes, cancer, cardiovascular diseases, hypertension, and psychiatric diseases, 7) no smoking, and 8) no history of head injury.

### Measures

#### Socio-demographic status

Demographic data including the health-related variables, history of fertility, and information about socio-demographic characteristics (age, marital status, occupation, educational status, etc.) were completed for all participants.

#### Psychological distress

The Beck Depression Inventory–Second Edition (BDI-II) is a 21-item self-report measure designed to measure severity of depression consistent with symptoms of depression (13). Moreover, the Persian version of BDI has been reported to be clinically appropriate for the assessment and research in the Iranian population by a validation study in Iran (14).

#### Cognitive performance

The Montreal Cognitive Assessment (MoCA) was developed by Ziad Nasreddine in Montreal (15). The MoCA assesses six cognitive domains including (1) executive functions (5 points); (2) visuospatial abilities (5 points); (3) delayed recall (short-term memory) (5 points); (4) language (3 points); (5) attention (6 points), concentration, and working memory (2 points); and (6) temporal and spatial orientation (6 points) (15). The psychometric properties of the questionnaire have been verified among the Iranian population (16).

#### Hirsutism

Ferriman and Gallwey Scoring System (F/G score) is a method of evaluating degree of hirsutism in females. In each of these areas, a score of 0 (absence of terminal hairs) through 4 (extensive terminal hair growth) was assigned. A score of eight or more was considered as positive for hirsutism according to the FG scoring system (17).

#### Acne

An assessment of acne was made using the Global Acne Grading System (GAGS). This system divides the face, chest and back into six areas (nose, forehead, chin, each cheek, chest and upper back). The six locations are graded separately on a 0–4 scores depending on the most severe lesion within that location (18).

#### Laboratory measures

We collected venous blood samples in third day of the follicular phase of the menstrual cycle after 8-hour fast early on the morning of the day. The total testosterone (TT) and sex hormone-binding globulin (SHBG) levels were measured by ECLIA (Cobas.e411. Roche, Germany). The free androgen index (FAI) can be used to estimate physiologically active testosterone in humans (19). This index is calculated as the ratio of TT divided by SHBG level (both expressed in the same units) and multiplied by 100 to yield numerical.

### Ethical consideration

This study was approved by the ethics committee of Tarbiat Modares University of Medical Sciences (IR.TMU.REC.1394.154). After explaining the study's purposes, written consent and verbal assent were collected from all participants who were informed that their participation was voluntary, confidential, and anonymous.

### Statistical analysis

The data were analyzed using the SPSS software (Statistical Package for the Social Sciences, SPSS Inc., Chicago, IL, USA), version 22.0. While the group comparisons were carried out using the Student's *t test*, Mann-Whitney U-test, and Chi-square test, the relationship between the variables, we determined using the Pearson's and Spearman's correlation Statistical coefficients. The statistical significance was set at P < 0.05.

## 3. Results

Table I describes the demographic characteristics of study participants and there were no significant difference between age, Body Mass Index (BMI), educational status, age, parity, occupation, and marital status (P > 0.05) (Table I).

The mean scores of clinical parameters such as hirsutisms and acne were evaluated in both groups. TT was significantly higher in the PCOS group. FAI showed a relatively significant difference between the two groups. Nevertheless, there was no significant difference in SHBG between the two groups. Moreover, we found a significant difference in depression and anxiety between the two groups (Table II).

The evaluation of the two groups with regard to MoCA showed that some mean values were statistically and significantly lower in the women in the case group. Besides, the differences of scores in the two groups were statistically significant in the domains of visual-spatial ability, executive function, attention, and total MoCA scores (Table III).

Table IV shows the association between acne, hirsutism, androgen hormone levels, and SHBG, depression and anxiety together with a total score of cognition and its domains. The results revealed that executive function was negatively associated with depression (p = 0.04). However, no correlations were detected between the MoCA components and TT, FAI, SHBG, acne, hirsutism, and anxiety in the case group (Table IV).

**Table 1:** Comparison of demographics characteristics between the two groups


**Variables**		**Case (n = 53)**	**Control (n = 50)**	**P-value**
Women age (yr)*		28.47 ± 6.27	29.94 ± 6.24	0.23
Parity*		0.84 ± 1	1.20 ± 1.18	0.13
BMI (kg/m${}^{2}$)*		28.74 ± 5.33	27.78 ± 4.45	0.32
Education (yr)*		11.49 ± 3.53	11.88 ± 3.69	0.58
Occupation**				
	**Employed**	37 (69)	40 (80)	0.47
	**Housewife**	9 (17)	5 (10)	
	**Student**	7 (13.2)	5 (10)	
Marital status**				
	**Unmarried**	7 (13.2)	7 (14)	0.92
	**Married**	43 (81.1)	41 (82)	
	**Divorced**	3 (5.7)	2 (4)	
* Data presented as Mean ± SD. Independent Student's *t* test; ** Data presented as n (%). Chi square, BMI: Body mass index				

**Table 2:** The mean scores of acne, hirsutism, total testosterone, FAI, SHBG, depression and anxiety between the two groups


**Variables**	Case	**Control **	**P-value**
**Acne score**	11.60 ± 6.89	8.64 ± 6.20	0.02
**Hirsutism score**	7.56 ± 6.52	2.63 ± 1.68	0.0001
**FAI**	1.26 ± 0.99	0.82 ± 0.69	0.009
**Total testosterone (nmol/L)**	0.41 ± 0.18	0.32 ± 0.16	0.008
**SHBG**	45.94 ± 29.64	51.68 ± 26.33	0.30
**Anxiety**	17.35 ± 10.44	12.20 ± 9.65	0.01
**Depression**	20.39 ± 9.85	14.46 ± 8.40	0.001
Data presented as Mean ± SD. *t* test, FAI: Free androgen index; SHBG: Sex hormone-binding globulin			

**Table 3:** Scores and total scores for the domain subgroups of MoCA between the two groups


**Variables**	Case	**Control **	**P-value**
**Total score**	22.83 ± 3.4	24.72 ± 2.52	0.002*
**Memory**	2.98 ± 1.49	3.2 ± 1.29	0.43*
**Visual-spatial ability**	3.30 ± 0.66	3.62 ± 0.60	0.009**
**Executive function**	2.71 ± 1.19	3.18 ± 1.02	0.05**
**Verbal performance**	3.20 ± 0.98	3.34 ± 0.96	0.64**
**Attention**	4.32 ± 1.54	4.98 ± 0.97	0.03**
**Orientation**	5.64 ± 0.52	5.64 ± 0.59	0.78**
Data presented as Mean ± SD. *Independent Student's *t* test; **Mann-Whitney U-test, MoCA: Montreal cognitive assessment			

**Table 4:** Correlation between acne, hirsutism, androgen hormone levels and SHBG, depression and anxiety with scores and total scores for the domain subgroups of MoCA


**Variables**		**Total score**	**Memory**	**Orientation**	**Attention**	**Verbal performance**	**Visual-spatial ability**	**Executive function**
**Acne**								
	**r**	-0.02**	0.01**	-0.025**	-0.066**	-0.16**	0.003**	-0.14**
	**p**	0.85	0.94	0.85	0.64	0.24	0.98	0.30
**Hirsutism**								
	**r**	-0.012**	-0.08**	-0.01**	-0.07**	0.01**	-0.01**	0.13**
	**p**	0.93	0.55	0.94	0.62	0.89	0.89	0.34
**Total testosterone**								
	**r**	-0.14*	0.005*	-0.04**	-0.20**	-0.16**	-0.26**	0.071**
	**p**	0.30	0.97	0.77	0.13	0.24	0.06	0.61
**SHBG**								
	**r**	-0.01*	0.02*	0.02**	-0.034**	0.018**	-0.13**	-0.03**
	**p**	0.94	0.84	0.88	0.81	0.89	0.35	0.82
**FAI**								
	**r**	-0.03*	0.000*	-0.03**	-0.078**	-0.05**	-0.05**	0.10**
	**p**	0.78	0.99	0.82	0.57	0.67	0.67	0.44
**Depression**								
	**r**	-0.03**	-0.061**	-0.02**	0.001**	-0.03**	-0.20**	-0.28**
	**p**	0.81	0.66	0.85	0.99	0.79	0.14	0.04
**Anxiety**								
	**r**	-0.15**	-0.23**	0.13**	0.05**	-0.079**	0.085**	-0.16**
	**p**	0.26	0.09	0.34	0.72	0.57	0.54	0.23
*Pearson correlation; **Spearman correlation, MoCA: Montreal cognitive assessment; SHBG: Sex hormone-binding globulin; FAI: Free androgen index; r: Correlation statistical coefficients, p-value consider significant when it < 0.05								

## 4. Discussion

### Acne and cognitive function

In the present study, there was a significant difference between the two groups in the mean levels of acne, hirsutism, total testosterone, free androgen index, depression, and anxiety. Some mean values of the MoCA were in the control group is higher than the case group. Additionally, the mean domains of visual-spatial ability, executive function, attention, and total MoCA scores between the two groups was significant difference.

Acne is a common skin condition in PCOS, which has a prevalence of 83% (7). The negative influence of acne on patients' psychological status that may impair cognitive function (20). We found some studies on cognitive function in patients with acne vulgaris. Recent studies showed improvements in cognitive abilities such as executive function, memory, attention, and hippocampal-based learning during isotretinoin therapy (21, 22). The hippocampus plays an important role in spatial and episodical memories associated with retinoid acid functions such as neural plasticity, neurogenesis, induction of expression receptor neurotrophin, promoting neuronal differentiation, and cell survival (7). In a retrospective cross-sectional study, the acne patients were affected twice more to Attention Deficit Hyperactivity Disorder (ADHD) compared to other patients (8). Since ADHD is associated with impaired cognitive functioning, including working memory, executive function, and attention, a higher level of ADHD in acne indicates a relationship between acne and cognitive impairment (23). Deveci and colleagues (6) studied the cognitive ability of 66 acne patients and 47 healthy controls. The acne group showed significantly worse performance in cognitive tests (learning, verbal fluency performance, and memory) compared with the control group (24). Oxidative stress can cause neurotoxicity through damage to DNA, lipid peroxidation, and neurotrophin reduction (25). Thus, perhaps, the two mechanisms for cognitive impairment in this disease are oxidative stress, anxiety and depression resulting from acne scars. In contrast to the aforementioned studies, our study found no association between acne and cognitive function.

### Depression, anxiety, and cognitive ability 

The findings of this study showed no connection between high levels of depression and anxiety in PCOS patients with cognitive impairment. In literature, prevalence of depression in women with PCOS is reported to be from 28 to 64%. High prevalence of anxiety was observed in PCOS was reported ranges from 34 to 57%. (26). The literature review showed cognitive defects (such as memory, attention, verbal performance, executive function, psychomotor function, and brain function) in the cognitive system of depressed and anxious people (11). Porter and cco-workers (26) found that about 50-75% of depressed patients have cognitive and memory impairment, called “pseudodementia depression." A review of studies revealed only three research projects with contradictory results. Kizilbash and co-authors (27) did not show any association between cognitive impairment and anxiety, while Beaudreau *et al* (28) indicated that depression alone was not related with any type of cognitive impairment. Therefore, the presence of both depressive and anxiety disorders will lead to cognitive impairment (29-30). A number of researchers have suggested that the association between anxiety and cognitive function is curved; they believe that anxiety at a mild level does not cause memory loss and can also improve cognitive function. Also, Christensen and Basso stated that people having more attacks of depression and anxiety are at higher risk of cognitive impairment (31). The subjects in the present study were young people (mean age = 29.18 yr) who had less experienced with depression and anxiety compared to the older individuals.

### Testosterone hormone and cognition

Our results indicated that there was no correlation between cognitive function and androgen levels. However, some studies have not found these associations (10, 31), but also others, have reported nearly linear (32) or nonlinear (33) relationships in this regard. The high testosterone levels might have a positive or negative impact on cognitive performance in women (9, 10). A large number of patients with PCOS have increased androgen levels (12). This disorder in androgen production by the ovaries appears at puberty but is rooted in childhood or embryonic period (34). Continuous exposure to testosterone before birth changes the cortical network related to cognitive ability (9). Nevertheless, in a review of studies, there are many contradictory results. Based on the study of Schattmann and colleagues (9), changes in the level of free testosterone had no significant effect on the cognitive function in women with PCOS. The study of Barry and co-workers (8) on 69 women with PCOS showed that a positive correlation exists between the three-dimensional mental rotation with testosterone levels. A study on healthy women, a single sublingual dose of 0.5 mg testosterone caused improvements in performance on object-location memory task (35) and visuospatial ability (36). Thrilers *et al* (36) showed circulating free testosterone levels were negatively correlated with verbal memory task and verbal fluency performance in women. Some studies (33, 37) have suggested a “curvilinear (inverted U-shape) relationship between serum testosterone levels and cognitive ability, whereby higher and lower levels of testosterone had low cognitive ability scores, whereas intermediate levels of testosterone were associated with better cognitive performance; these findings suggest that a the level of serum testosterone in mid-range may be responsible with optimal cognitive performance”.

In the present study, the cognitive function of the patients was weaker than that of the healthy group, but no relationship was found between the mild cognitive impairment (MCI) and the level of hormones, which could be due to:

a) Small sample size;

b) Increase in the level of total and free testosterone, which was higher in the patient group than in the healthy subjects but was in the normal range;

c) Duration of exposure to hyperandrogenism: As observed, the mean age of the participants was 28-29 yr, so they are less exposed to hyperandrogenism than the older population. The duration of the disease may be effective; and

d) In addition to testosterone, other hormones such as estrogen, LH, FSH, progesterone, prolactin, and insulin, either alone or in combination, can be effective in the cognition functions of these patients. To date, the effect of these hormones on cognition functions, especially in patients with PCOS has not been reported.

It should also be taken into account that the effect of androgen hormones on cognitive functions is not influenced only by the amount of testosterone, rather the activity of androgen receptors and the metabolic activity of androgen are effective as well, which, in turn, are dependent to a certain degree on genetic factors.

### PCOS and cognition

In the present study, the prevalence of MCI (scores < 26) was 81.1% in the patients and 54% in the control groups. Moreover, there was a significant difference in the mean levels of cognitive functioning between groups. This difference was in the form of a decrease in cognitive performance (total) in the case group compared to the control group. To the best of our knowledge, there is limited research in the field of cognitive function in patients with PCOS. The results of Barnard and colleagues (1), and Ghazeeri *et al* (37) showed that PCOS women have poor performance in some domains of cognition. Although the patient's group showed poorer performance on executive ability, visual-spatial function, and attention, there was no correlation between this defect and the considered variables. Cognitive impairment could be due to obesity (38), insulin resistance (39), and increased estrogen hormones (40), etc. This research is the first cohort study in Iran that examined the relationship between these variables and cognitive impairment in PCOS women at the same time. According to the information obtained by performing a comprehensive review, it is a rare study about cognitive impairment in patients with PCOS. We suggest further studies with larger sample numbers and different age groups to assess cognitive function in women as well as to find the causes of cognitive impairment in these patients.

## 5. Conclusion

The present study demonstrated that the PCOS patients had significantly poorer performance on the tests of verbal fluency, attention, and visual-spatial function than the healthy control women. No differences between the two groups were found on the tests of memory, orientation, and executive function. Also, there was no correlation between this defect and levels of free testosterone, TT, SHBG, acne, hirsutism, depression, and anxiety.

##  Conflict of Interest

The authors declare that there is no conflict of interest.
